# Impact of habitual physical activity and type of exercise on physical performance across ages in community-living people

**DOI:** 10.1371/journal.pone.0191820

**Published:** 2018-01-25

**Authors:** Francesco Landi, Riccardo Calvani, Anna Picca, Matteo Tosato, Anna Maria Martone, Emanuela D’Angelo, Elisabetta Serafini, Roberto Bernabei, Emanuele Marzetti

**Affiliations:** Department of Geriatrics, Neurosciences and Orthopedics, Teaching Hospital "Agostino Gemelli", Catholic University of the Sacred Heart, Rome, Italy; Universita degli Studi di Napoli Federico II, ITALY

## Abstract

The maintenance of muscle function into late life protects against various negative health outcomes. The present study was undertaken to evaluate the impact of habitual physical activity and exercise types on physical performance across ages in community-living adults. The Longevity check-up 7+ (Lookup 7+) project is an ongoing cross-sectional survey conducted in unconventional settings (e.g., exhibitions, malls, and health promotion campaigns across Italy) that began on June 1^st^ 2015. The project was designed to raise awareness in the general population on major lifestyle behaviors and risk factors for chronic diseases. Candidate participants are eligible for enrolment if they are at least 18 years of age and provide written informed consent. Physical performance is evaluated through the 5-repetition chair stand test. Analyses were conducted in 6,242 community-living adults enrolled between June 1^st^ 2015 and June 30^th^ 2017, after excluding 81 participants for missing values of the variables of interest. The mean age of the 6,242 participants was 54.4 years (standard deviation 15.2, range 18–98 years), and 3552 (57%) were women. The time to complete the chair stand test was similar from 18 to 40–44 years, and declined progressively across subsequent age groups. Overall, the performance on the chair stand test was better in physically active participants, who completed the test with a mean of 0.5 s less than sedentary enrollees (p < .001). After adjusting for potential confounders, a different distribution of physical performance across exercise intensities was observed, with better performance being recorded in participants engaged in more vigorous activities. Our findings suggest that regular physical activity modifies the age-related pattern of decline in physical performance, with greater benefits observed for more intensive activities. Efforts are needed from health authorities and healthcare providers to promote the large-scale adoption of an active lifestyle throughout the life course.

## Introduction

The demographic transition experienced by developed countries over the last decades poses unprecedented challenges for managing the care of older persons. As a result, there is growing demand for devising effective solutions against the detrimental consequences imposed on healthcare systems by age-related conditions (in particular, disabilities) [1,2]. In this context, sarcopenia (i.e., the age-dependent loss of muscle mass and strength/function) has gained special interest. Indeed, this condition, while conferring greater risk of physical function decline, disability and mortality, is potentially preventable [3,4].

Recently, we explored changes of muscle mass and function across ages in an unselected sample of community-dwelling persons [5]. Overall, muscle mass slightly decreased with advancing age, while physical performance (as assessed through the chair stand test) remained stable during the first decades of adulthood, and decreased at middle age (45+) and late adulthood. Noticeably, individuals older than 75 displayed 30% reduction of physical function compared with the youngest group [5]. Apart from age, sedentary lifestyle is a major factor contributing to muscle weakness which, in turn, results in reduced activity levels and further loss of muscle mass and function [6]. With the notable exception of professional athletes [7,8], it is unclear whether age-related patterns of muscle function decline may be substantially modified by an active lifestyle in the general population.

The present study was, therefore, undertaken to explore the relationship between habitual physical activity and age-related changes in muscle function across ages (from 18 to 98 years) in an unselected sample of community-dwellers enrolled in the Longevity Check-up 7+ (Lookup 7+) project. We also sought to determine if distinct types of activity could differentially impact physical performance parameters.

## Methods

### Ethics statement

Prior to enrollment in the study, all participants provided written informed consent. In no case informed consent was obtained from next of kin, carers or guardians on the behalf of participants. The study was conducted according to the principles expressed in the Declaration of Helsinki. The protocol was approved by the Catholic University of the Sacred Heart Ethics Committee.

### Study population

The Lookup 7+ project is an ongoing initiative developed by the Department of Geriatrics of the Catholic University of the Sacred Heart (Rome, Italy). The project started on June 1^st^ 2015 and was designed to promote the adoption of healthier lifestyles by raising awareness in the general population on major lifestyle behaviors and risk factors for chronic diseases. A dedicated team of medical doctors, researchers, and nutritionists assesses people visiting public places (e.g., malls, exhibition centers) and those adhering to prevention campaigns launched by our department. This approach has been chosen because allowing for enrolling relatively unselected participants, outside of conventional healthcare or research settings. Candidate participants are considered to be eligible for enrolment if they are at least 18 years of age and provide written informed consent. Self-reported pregnancy, inability to perform functional tests, and unwillingness to give written informed consent are considered exclusionary.

For the present study, analyses were conducted on data collected between June 1^st^ 2015 and June 30^th^ 2017. Participant recruitment took place in the following settings: *Milan EXPO* 2015 (Milan, June-October 2015), *Mese del Cuore* (Rome, October-September 2016), *La Romanina—Check your Longevity* (Rome, December 2017), *Mese del Cuore* (Milan, March-April 2017), Ministry of Health—Women’s Day (Rome, April 2017), *CamBio Vita* (Catania, May 2017), *COOP* shopping centers (Bologna, Modena, Genoa, Rimini, and Grosseto, May-June 2017). Depending on the setting, the initiative was advertised in newspapers, magazines and TV broadcasting. Visitors were also invited to participate by direct contact. During this 2-year time-frame, 6,323 community-dwellers were enrolled in the Lookup 7+ project. However, 81 participants were excluded from the present analyses for missing values of the variables of interest; hence, the study population consisted of 6,242 people.

All people who accepted to be assessed underwent individual evaluations consisting of a brief questionnaire and the measurement of cardiovascular health metrics, anthropometric parameters, and physical performance. In particular, the Lookup 7+ visit was structured to collect the following information and data: written informed consent, lifestyle interview (smoking and eating habits, habitual physical activity), blood pressure, weight and height, total blood cholesterol and glucose, and lower extremity muscle function. At the end of the assessment, participants were provided with their cardiovascular health metrics score along with suggestions on how to improve/change their lifestyle and on the eventual need for further assessments [9].

Smoking habit was categorized as current or never/former smoker. Standing height was measured using a standard stadiometer. Body weight was measured through an analogue medical scale. Body mass index (BMI) was calculated as the weight (kg) divided by the square of height (m). Healthy diet was defined as the consumption of at least three portions of fruit and/or vegetables per day [10]. For the calculation of daily intake of fruit and vegetables, we used the reference tables for the Italian population released by the Italian Society of Nutrition (SINU). Accordingly, three or more portions of fruit and/or vegetables correspond to more than 400 g, which is the minimum amount recommended by the World Health Organization. The use of three or more portions to identify a healthy diet is in line with Italian dietary habits for fruit and vegetables which are typically eaten during the main meals rather than as snacks. Reference amounts are available at http://www.sinu.it/html/cnt/larn.asp. Total blood cholesterol was measured from capillary blood samples using disposable reagent strips based on a reflectometric system with the portable device MultiCare-In (Biomedical Systems International srl, Florence, Italy) [11]. The same device was employed to measure random blood glucose using reagent strips based on an amperometric system [11]. Blood pressure was measured with an electronic sphygmomanometer following the recommendations from international guidelines [12].

### Habitual physical activity

Regular participation in physical activity was operationalized as the involvement in physical activity at least twice weekly during the last year. Accordingly, participants were categorized as sedentary or physically active. To be assigned to the active group the following activities were considered: light walking for at least 30 min per session (leisure walking), cycling, swimming, running, and resistance training. An *ad hoc* variable was created considering five groups: 1) sedentary (n = 3,331); 2) leisure walking (n = 1,277); 3) aerobic training (cycling, swimming, and running) (n = 975); 4) resistance training (n = 396); and 5) combination of aerobic and resistance training (n = 263).

### Outcome assessment

Physical performance was evaluated by means of the chair stand test. This test is part of the Short Physical Performance Battery (SPPB) [13]. Participants were asked to stand up from a chair with their arms folded across the chest five times in a row as quickly as possible. A standard 43-47-cm-high armless chair was used. The time taken to perform the task was measured using a handheld stopwatch. The chair stand test was chosen because it is a simple-to-use, reliable and valid measure of physical function in adult and older people, including those with musculoskeletal or neurological conditions. Poor performance on this test is suggestive of mobility problems and is associated with greater risk of developing disability [14]. Previous studies have shown that the chair stand test is highly reliable [intraclass correlation coefficients (ICCs) 0.76–0.99 for test-retest reliability and ICCs 0.97–1.00 for inter-rater reliability] [15,16]. Compared with upper extremity strength measures (e.g., handgrip strength test), the chair stand test offers several advantages: (1) it focuses on the performance of lower extremities which is more critical to functional performance and declines more dramatically during aging than upper extremity function; (2) it provides a measure of muscle power which is a stronger predictor of functional performance and is less dependent on muscle mass than strength; (3) it offers information that goes beyond muscle function (e.g., ability to coordinate multiple body segments and to execute a multistep task) (reviewed in [17]).

### Statistical analysis

Continuous variables are expressed as mean ± standard deviation (SD), whilst categorical variables are shown as absolute numbers and percentages. Descriptive statistics were used to assess demographic and main clinical characteristics of the study population according to physical activity status. Differences in proportions and means of covariates among physical activity groups were assessed using the Fisher’s exact test and the t-test, respectively.

Physical activity was considered as the independent variable and physical performance (chair stand test) was used as the dependent variable. Analysis of covariance (ANCOVA) was used to examine the association between different types of activities and physical performance levels. Assumption of normality was verified by checking the histogram of the dependent variable and that of residuals. Homogeneity of variances for the independent variables was established using the Levene’s test of equality of error variances. The plot of residuals against the identification observation number was used to check independence of observations. Variables considered for adjustment were those showing statistically significant different distribution across physical activity groups at the univariate analysis (i.e., age, gender, smoking habit, healthy diet, BMI, systolic blood pressure, and cholesterol).

Logistic regression analysis was used to assess the association between different types of physical activity and the performance on the chair stand test. To this aim, participants were classified in two groups according to gender-specific chair stand test median values (7.59 s and 7.40 s for women and men, respectively). All variables associated with physical activity status at a significance level of *p* < .05 at the univariate analysis were entered in the models. To identify factors independently associated with lower performance, we first estimated the crude prevalence rate ratio at 95% confidence interval (CI), and then controlled for age and gender. Final analyses were therefore adjusted for age and gender (model 1); age, gender, smoking habit, and healthy diet (model 2); age, gender, smoking habit, healthy diet, systolic blood pressure, and total blood cholesterol (model 3). Age, systolic blood pressure and blood cholesterol were treated as continuous variables.

The focus of the analytic plan was also to explore age-related trends of physical performance across physical activity groups. For this reason, the study sample was categorized in the following age groups: <24, 25–29, 30–34, 35–39, 40–44, 45–49, 50–54, 55–59, 60–64, 65–69, 70–74, 75–79, and 80+ years.

All tests were 2-sided with statistical significance set at *p* < .05. All analyses were performed using the SPSS software (version 11.0, SPSS Inc., Chicago, IL).

## Results

The mean age of the 6,242 participants included in the analyses was 54.4 years (SD 15.2, range 18–98), and 3,552 (57%) were women. The main characteristics of the study population according to physical activity status are summarized in Table 1. As compared with sedentary participants, those who were physically active showed higher prevalence of healthy diet and lower BMI (*p* < .001). Systolic blood pressure and total blood cholesterol were lower in the physically active group (*p* = .01). The performance on the chair stand test was significantly better in physically active participants, who completed the test on average 0.5 s less than sedentary enrollees (*p* < .001).

**Table 1:** Characteristics of the study population according to physical activity*.

Characteristics	Total sample (n = 6,242)	Physically active (n = 2,911)	Sedentary (n = 3,331)	*p*
**Age** (years)	54.4 ± 15.2	53.9 ± 15.3	54.7 ± 15.1	.09
**Gender** (female)	3,552 (57)	1,549 (53)	2,003 (60)	< .001
**Active smoking**	1,024 (16)	436 (15)	588 (18)	< .01
**Healthy diet**	4,499 (73)	2,276 (79)	2,223 (67)	< .001
**BMI** (kg/m^2^)	25.4 ± 4.3	24.9 ± 3.9	26.1 ± 4.5	< .001
**Systolic BP** (mmHg)	126 ± 17	125 ± 17	126 ± 17	.01
**Diastolic BP** (mmHg)	76 ± 10	76 ± 9	76 ± 10	.11
**Cholesterol** (mg/dL)	211 ± 35	209 ± 35	212 ± 36	< .01
**Glucose** (mg/dL)	103 ± 23	103 ± 23	103 ± 24	.64
**Chair stand test** (s)	7.7 ± 2.1	7.4 ± 1.9	7.9 ± 2.3	< .001

* Data are given as number (percentage) for gender, smoking, and healthy diet; for all the other variables, means ± standard deviations are reported.

Regular physical activity: physical activity at least twice weekly during the past year.

Healthy diet: consumption of at least three portions of fruit and/or vegetables per day.

BMI: body mass index; BP: blood pressure

**Fig 1:**
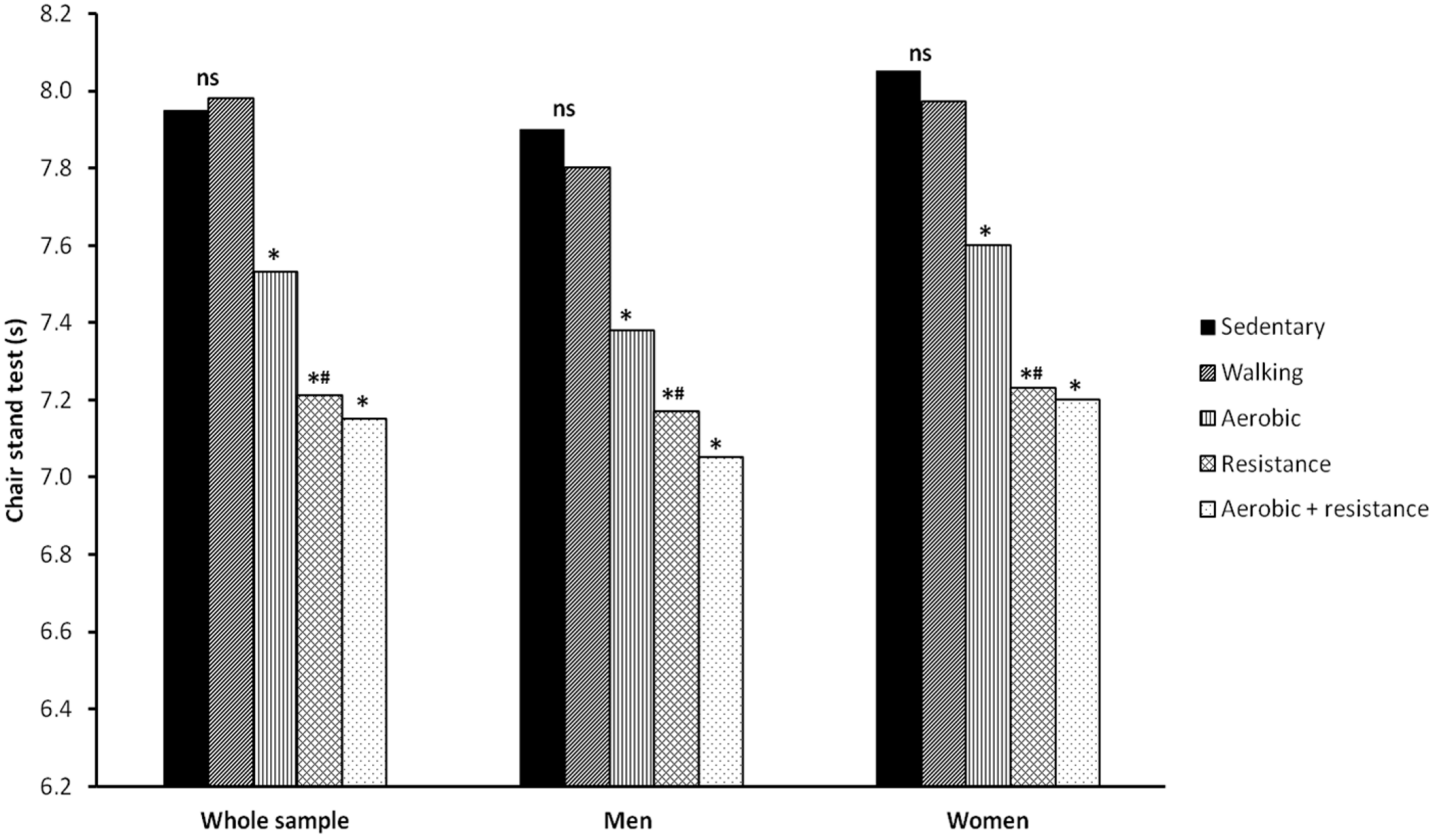
Time to complete the chair stand test (s) according to physical activity status and adjusted for age, gender, smoking habit, healthy diet, body mass index, systolic blood pressure, and total blood cholesterol. Physical performance, as assessed by the chair stand test, was comparable between sedentary participants and those practicing leisure walking only. The time taken to complete the test was shorter across increasing activity intensities, from aerobic training to combination of aerobic and resistance exercise. Walking vs. sedentary group, *p* = ns; aerobic activity vs. sedentary and walking group, *p* < .0001 (*); resistance activity vs. sedentary and walking group, *p* < .001 (*); resistance versus aerobic activity, *p* = .01 (#); combination of aerobic and resistance activity vs. sedentary and walking group, p < .001 (*).

Adjusted results from ANCOVA models showing the differences of chair stand test results across physical activity groups are shown in Fig 1. After adjusting for age, gender, smoking habit, healthy diet, systolic blood pressure, and cholesterol levels, a significant different distribution of physical performance across increasing activity intensities was observed. Indeed, as the activity intensity increased—from leisure walking to resistance exercise—physical performance improved significantly. In particular, participants practicing resistance training showed a better performance of about 0.8 s relative to their sedentary counterparts.

The association between habitual physical activity and better performance on the chair stand test (lower than median value) was also explored using specific multivariate models. As shown in Table 2, after adjustment for potential confounders, which included age, gender, smoking habit, healthy diet, systolic blood pressure and cholesterol levels, participants practicing a combination of aerobic and resistance training showed a higher probability of better physical performance (odds ratio 2.45; CI 1.74–3.45). No significant interaction was determined between age and physical activity (odds ratio 1.00; CI 0.99–1.01).

**Table 2:** Unadjusted and adjusted association [odds ratio (OR) and 95% confidence intervals (CIs)] between types of physical activity and better performance on the chair stand test (lower than median value).

	*Univariate OR* (95% CI)	*Adjusted OR Model 1* (95% CI)	*Adjusted OR Model 2* (95% CI)	*Adjusted OR Model 3* (95% CI)
**Physical activity status**				
Sedentary	1.0 (Reference)	1.0 (Reference)	1.0 (Reference)	1.0 (Reference)
Leisure walking	0.85 (0.75–1.01)	1.08 (0.94–1.24)	1.07 (0.93–1.24)	1.06 (0.91–1.23)
Aerobic activity	1.91 (1.64–2.21)	1.70 (1.45–1.99)	1.67 (1.42–1.96)	1.63 (1.38–1.92)
Resistance activity	2.29 (1.84–2.85)	2.10 (1.65–2.66)	2.08 (1.63–2.64)	2.19 (1.70–2.81)
Aerobic + resistance	3.51 (2.58–4.78)	2.39 (1.73–3.32)	2.40 (1.72–3.35)	2.45 (1.74–3.45)

**Model 1**: adjusted for age and gender.

**Model 2**: adjusted for age, gender, smoking habit, and healthy diet.

**Model 3**: adjusted for age, gender, smoking habit, healthy diet, systolic blood pressure, and cholesterol levels.

**Fig 2:**
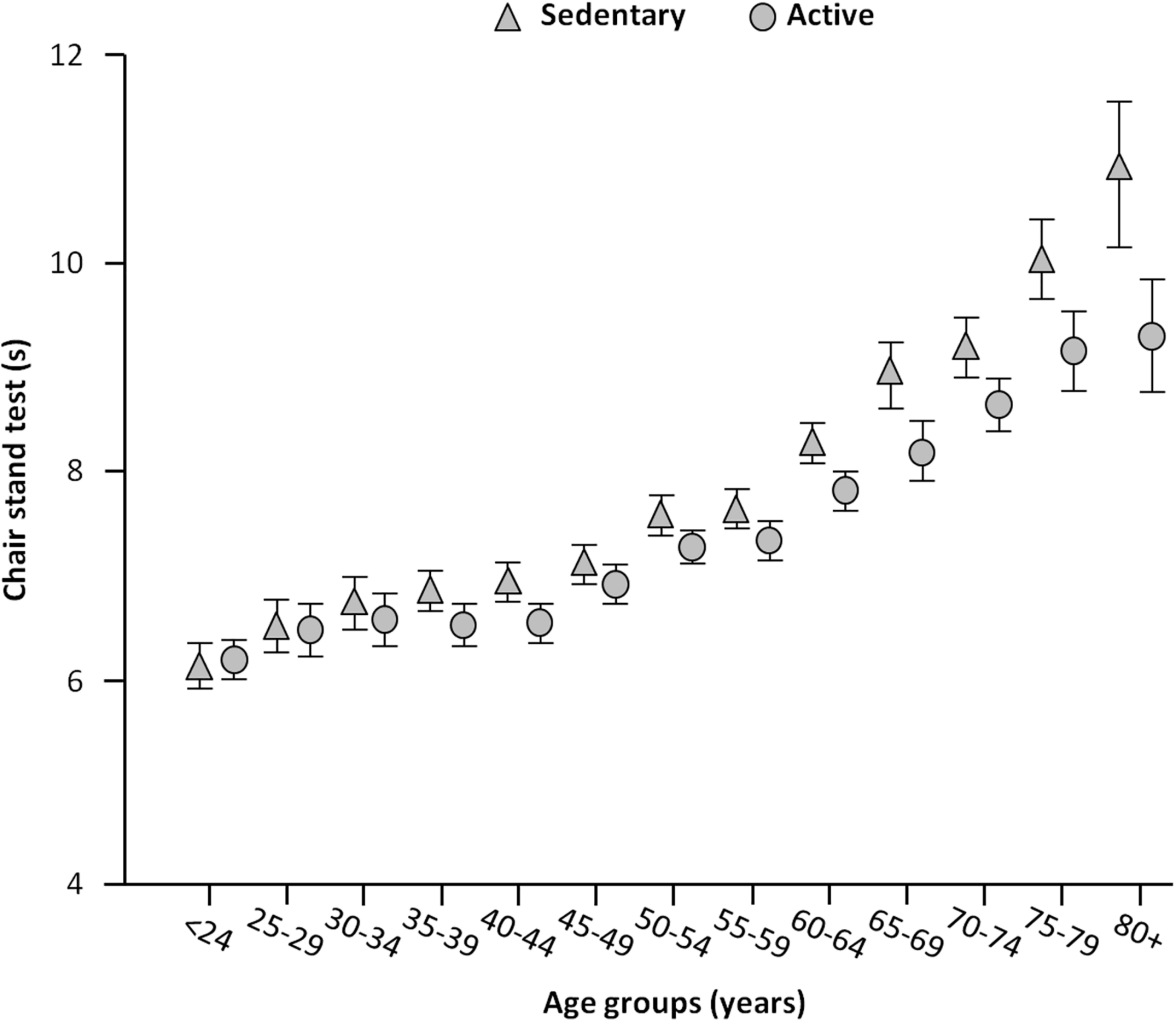
Time to complete the chair stand test (s) according to age groups and physical activity status. Physical performance, as assessed by the chair stand test, was similar from 18 to 40–44 years, and declined thereafter. Physically active participants showed better performance than sedentary people. Active 80+ participants showed the same performance of sedentary 65-year-old persons.

Fig 2 shows means and SDs of physical performance across age groups and according to physical activity. Overall, the time to complete the chair stand test was similar from 18 to 40–44 years, and declined linearly thereafter, such that the time taken to complete the test increased by more than 3 s from the youngest (<24 years) to the oldest group (80+ years). However, physically active participants showed better performance than sedentary people with a noticeable change in the pattern of decline more evident past the age of 55. Remarkably, active 80+ participants showed the same performance of sedentary 65-year-old persons.

**Fig 3:**
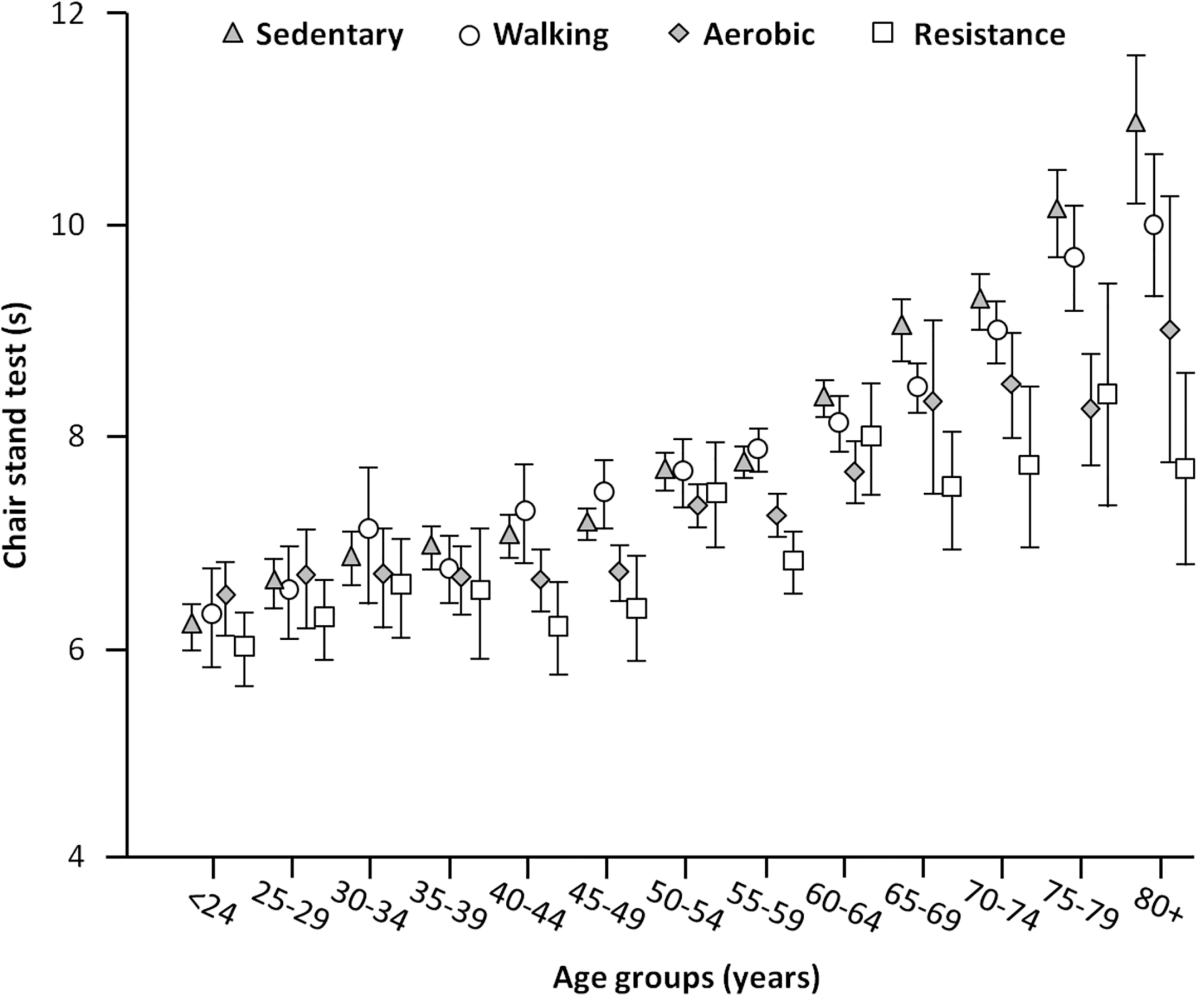
Time to complete the chair stand test (s) according to different types of physical activity and age groups. The age-dependent decline in physical performance was similar between sedentary participants and those practicing only leisure walking. The rate of decline was significantly attenuated in participants engaged in aerobic or resistance training Ultra-octogenarians practicing resistance training performed similar to 50-54-year-old participants.

Finally, to further substantiate the benefits of physical activity, we analyzed the impact of different types of activities on the age-related decline in physical performance (Fig 3). Participants practicing only leisure walking showed the same decline observed in the sedentary group. In contrast, those engaged in aerobic or resistance training displayed a clear difference relative to the inactive group. Specifically, 80+ participants practicing aerobic activities showed the same performance recorded in the 70-74-year group. Strikingly, ultra-octogenarians engaged in resistance training performed similar to 50-54-year-old enrollees.

## Discussion

The present study, conducted as part of the Lookup 7+ project, examined a large and unselected cohort of Italian community-dwelling men and women ranging in age between 18 and 98 years. To the best of our knowledge, our survey is the first assessing age-related changes of muscle function in a general and non-selected population. In this respect, the Lookup 7+ project provided the unique opportunity of assessing this parameter among people of both genders across a wide age range, outside of conventional healthcare or research settings. Using this innovative database, the present study shows that physical activity has a remarkable impact on physical performance in individuals living in the community, independent of gender and other variables. Indeed, after adjusting for several confounders, such as age, gender, smoking habit, healthy diet, blood pressure and cholesterol levels, better physical performance was more frequently observed in the highest level of physical activity (combination of aerobic and resistance training). More specifically, physical activity and, in particular, resistance training seem to be able to modify the age-related decline of physical performance. Strikingly, oldest participants engaged in resistance exercise training performed the chair stand test in a time comparable to that of sedentary middle-aged enrollees. In other words, practicing resistance exercise into old age seems to hold back the "physical performance clock" by approximately 30 years.

Our data are consistent with previous studies showing that people who are more physically active have a reduced risk to develop mobility disability compared with those who are less active [18–21]. These findings corroborate the knowledge in this field and also show that the age-related decline in physical performance is potentially amendable. Nevertheless, most of the available evidence was obtained in selected population (e.g., frail older people, professional/master athletes), specific clinical settings (e.g., outpatient clinics), or following short-term exercise training protocols [3,7,8,22]. Our results obtained in an unselected population lend further support to the importance of implementing interventions at relatively young age in order to prevent, attenuate, or delay functional decline in late life [23,24]. Efforts to preserve strength and endurance should begin at middle age, in order to ensure sufficient functional reserve later in life [25]. Such a primary prevention approach may be based on the prescription of specific training programs and nutritional interventions [10,26].

Physical inactivity is a major causative factor for muscle loss and weakness which, in turn, results in further deconditioning, exacerbation of muscle wasting, and increased fatigability [24]. Instead, physical exercise, being highly effective at counteracting the decline in muscle mass and function associated with aging, is considered to be the most effective strategy against sarcopenia [27].

Engagement in regular physical activity conveys a wealth of health benefits in young and old populations [28]. Indeed, the World Health Organization recommends people of all ages being as physically active as possible and including at least 150 min a week of moderate-intensity physical activity (e.g., brisk walking) or 75 min of vigorous-intensity activity (e.g., running) throughout the week [29]. Additional benefits may be achieved by doubling the amount of physical activity [29]. Activity levels below those recommended have still been associated with reduced risk of negative health outcomes [30]. Yet, our results indicate that leisure walking confers only a marginal effect on physical performance compared with higher intensity exercises (strength training) and/or a combination of different exercises (aerobic and strength training).

Completing the five-repetition chair stand test requires considerable skill to generate sufficient speed of movement and coordinate multiple segments with correct timing in order to maintain balance. Hence, the time taken to complete this task is likely determined by factors such as coordination and disease-specific impairments [14]. Poor performance on this test highlights mobility problems and is associated with higher risk of subsequent disability [13,14]. Moreover, the chair stand test shows good correlation with gait speed, balance, and knee extensor strength. Our finding that specific exercise trainings (aerobic and resistance activities) are able to maintain better performance on the chair stand test during the life course implies that such training modalities may prevent mobility disability late in life.

Albeit dealing with a highly relevant issue, our study presents some limitations that need to be discussed. First, the evaluation setting could have influenced the assessment of physical performance. Although the chair stand test was assessed according to the standard protocol [13], participants were involved—before the evaluation—in usual activities, such as walking, carrying bags and eating, which might have influenced the test results. In particular, the chair stand test in older individuals might have been more affected by tiring activities than in younger participants. Second, the results shown in this paper were obtained from a cross-sectional survey. Hence, findings could be influenced by differences in the birth cohort. For example, the prevalence of physical activity as well as other domains considered (i.e., smoking and healthy diet) could be related to an intrinsic attitude of different birth cohorts and not be associated to actual age-related changes. Furthermore, it is possible that unmeasured differences exist between activity groups. For instance, participants reporting higher levels of physical activity may also have higher levels of medical care. However, our homogeneous population of unselected community-living people minimizes the possibility that enrollees who reported higher physical activity levels had substantially better healthcare or health knowledge than those with sedentary lifestyle. It should also be considered that, given the cross-sectional design of our study, reverse causality between physical activity and muscle function cannot be ruled out. Indeed, it is possible that lack of physical activity was due to pre-existing functional limitations rather than vice versa. Because frequency or volume of physical activity was not collected, a dose-effect relationship with physical performance cannot be established. Finally, the Lookup 7+ study population included only Caucasian persons, which impedes the generalizability of our results to other ethnic groups.

## Conclusions

Our study offered the unique opportunity of investigating age-related changes in physical performance and their relationship with physical activity in a large sample of unselected community-dwellers. Physical performance assessed by means of the chair stand test considerably declined with aging. Yet, physical activity, independent of gender and other variables, modified the pattern of decline. In particular, aerobic and resistance activities showed the higher positive impact on physical performance. This observation strengthens the evidence that regular exercise is effective at improving physical performance in adult and old persons. Therefore, health authorities and healthcare organizations, together with healthcare providers, should encourage all persons to be more physically active even during the extreme ages of life. A deeper understanding of the mechanisms that impair muscle performance with aging may reveal additional targets for intervention aimed at improving mobility and preventing physical disability in late life.
